# From seeds to symptoms: the molecular landscape of tau seeding in Alzheimer’s disease

**DOI:** 10.3389/fnins.2026.1927017

**Published:** 2026-09-17

**Authors:** Jin Miao, Chunhong Xue, Xuchao Yan, Jianlan Gu, Dandan Chu, Huaichang Sun

**Affiliations:** 1 College of Veterinary Medicine (Institute of Comparative Medicine), Yangzhou University, Yangzhou, Jiangsu, China; 2 Laboratory Animal Center, Nantong University, Nantong, Jiangsu, China; 3 Key Laboratory of Neuroregeneration of Jiangsu and Ministry of Education, NMPA Key Laboratory for Research and Evaluation of Tissue Engineering Technology Products, Co-Innovation Center of Neuroregeneration, Nantong University, Nantong, Jiangsu, China; 4 Department of Biochemistry and Molecular Biology, School of Medicine, Nantong University, Nantong, Jiangsu, China

**Keywords:** Alzheimer’s disease, extrinsic factors, intrinsic factors, mutation, prion, tau

## Abstract

Alzheimer’s disease (AD) is the leading cause of dementia in the elderly, pathologically defined by extracellular amyloid-β plaques and intracellular neurofibrillary tangles (NFTs) composed of hyperphosphorylated tau. The Braak staging system established that tau pathology spreads in a stereotypical pattern across the brain, supporting the “prion-like” hypothesis: misfolded tau aggregates serve as seeds, inducing the misfolding and aggregation of normal tau, and thereby driving disease progression along neuroanatomical pathways. In the past 5 years (2021–2026), substantial advances have elucidated the molecular mechanisms underlying tau seeding activity. This review summarizes recent progress in tau seeding research, highlighting both intrinsic and extrinsic regulators of tau prion-like activity. Intrinsic factors include the structural basis of tau strains, MAPT mutations, alternative splicing (3R/4R isoforms), post-translational modifications, and liquid–liquid phase separation. Extrinsic factors encompass cofactors, molecular chaperones, non-coding RNAs, epigenetic mechanisms, environmental exposures, and the emerging concept of trans-seeding by heterologous protein fibrils. We also review therapeutic strategies targeting tau pathology, focusing on their mechanisms and clinical development. Integrating these mechanistic insights and therapeutic advances will inform future research and clinical strategies for AD and related tauopathies.

## Introduction

Alzheimer’s Disease (AD) is the most prevalent neurodegenerative disorder among the elderly. Its hallmark symptoms include a progressive decline in learning and memory, along with a range of dementia-related issues such as aphasia, apraxia, agnosia, and changes in personality and behavior. AD accounts for about 60%–70% of all dementia cases, placing significant burdens not only on patients’ families but also on public health systems (Jia et al., 2020). AD is characterized by two primary neuropathological features: (1) extracellular amyloid plaques formed from aggregated β-amyloid (Aβ) and (2) intracellular neurofibrillary tangles (NFTs), which are primarily composed of abnormally hyperphosphorylated tau protein. Research indicates that the quantity of NFTs, rather than the number of amyloid plaques, is correlated with the severity of dementia in AD patients (Iqbal et al., 2018).

Prior to experimental confirmation, scientists noticed that tau pathology in AD follows a consistent pattern. The Braak staging system, created in the early 1990s, describes how tau NFTs spread from the locus coeruleus and entorhinal cortex to the limbic system and neocortex, following a stereotypical pattern, supporting the concept of “pathological propagation” along neuroanatomical pathways (Braak and Braak, 1991, 1995). In the late 1990s, researchers began to identify a connection between progressive central nervous system disorders, including AD, Parkinson’s disease (PD), and certain prion diseases such as Creutzfeldt-Jakob disease (CJD). These conditions are all linked to the misfolding, aggregation, and deposition of proteins (Carrell and Gooptu, 1998; Walker and LeVine, 2000; Skovronsky et al., 2006). The concept of tau having “prion-like” behavior emerged, suggesting tau and Aβ could act as “proteopathic seeds” that induce normal proteins to misfold and spread pathology between cells (Jucker and Walker, 2013; Goedert, 2015). In 2009, Clavaguera et al. showed that tau aggregates injected into mouse brains could induce misfolding of normal tau in distant brain areas, proving tau’s seeding and propagation ability *in vivo* (Clavaguera et al., 2009). Later studies using tau from human AD brains confirmed these findings (Sanders et al., 2014; Boluda et al., 2015; Kaufman et al., 2016) and led to the “tau strain hypothesis (Hyman, 2014; Goedert et al., 2017; Ayers et al., 2018),” which proposes that different tau aggregate conformations cause different tauopathies, explaining the clinical diversity of these diseases.

Understanding how tau protein spreads within the brain is essential for comprehending the progression of AD from localized pathology to widespread cognitive impairment. This article aims to primarily systematically review the latest advances in tau seeding research over the past 5 years (from January 1, 2021, to June 1, 2026). It will explore the influence of the biochemical properties of tau seeds as well as the host microenvironment on tau seeding, and conclude with current therapeutic strategies that target tau seeding in the context of AD. Finally, this review will discuss remaining challenges and considerations for future foundational research and clinical applications.

## Pathological basis of tau seeds

### Tau seeds: the nature of the pathogenic unit

Human microtubule-associated protein tau, encoded by the *MAPT* gene on chromosome 17q21.31, physiologically promotes microtubule assembly and stabilizes the microtubule network (Iqbal et al., 2016). Full-length central nervous system tau, also known as Tau441, consists of 441 amino acids and has a molecular weight of approximately 46 kDa. It features four distinct domains: (i) an N-terminal acidic projection domain (M1–Y197) that extends away from the microtubule surface, (ii) a proline-rich and basic region (I151–Q244) essential for interactions with SH3-domain-containing proteins, including proline-directed kinases, (iii) the microtubule-binding repeats (MTBRs) (T245–N368) that facilitate microtubule assembly and promote self-aggregation, and (iv) a C-terminal sequence (K369–L441) (Chu et al., 2023; Figure 1A). In the central nervous system (CNS), the *MAPT* gene produces six tau isoforms that vary in their N-terminal inserts and contain either three (3R-Tau) or four (4R-Tau) microtubule-binding repeats (Chu and Liu, 2019). Native tau monomers are predominantly intrinsically disordered, existing in highly flexible random coil conformations. However, they can transiently adopt a ‘paperclip’-like structure where the acidic N- and C-termini fold back over the basic MTBRs, effectively inhibiting self-aggregation (Jeganathan et al., 2006; Mandelkow et al., 2007).

**FIGURE 1:**
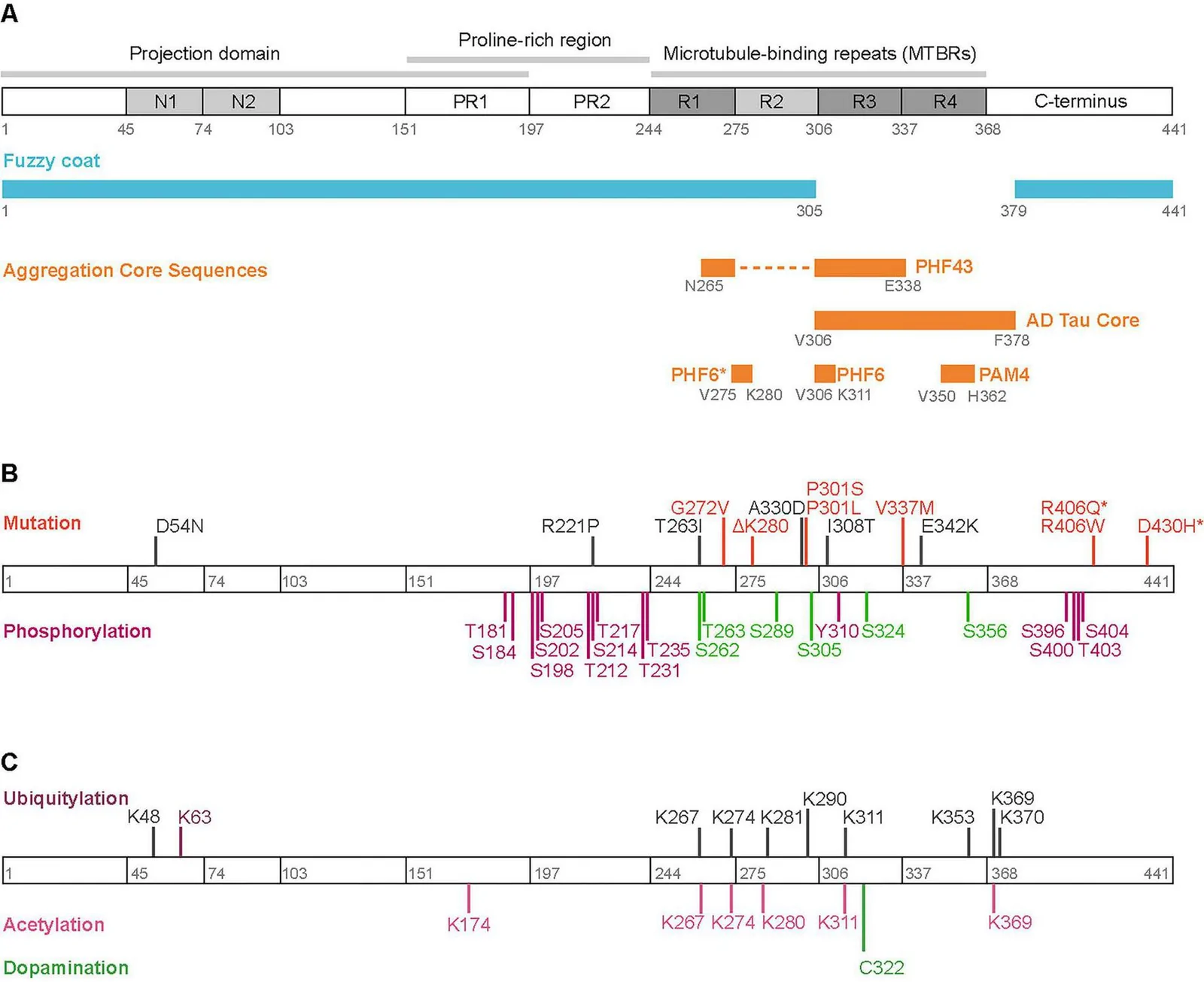
Schematic representation of tau protein structure and major PTMs. **(A)** The top panel shows the primary structure of the tau protein (441 amino acid isoform), with key domains labeled: projection domain, proline-rich regions (PR1 and PR2), microtubule-binding repeats (MTBRs: R1–R4), and the C-terminus. The “Fuzzy coat” region and various reported “Aggregation Core Sequences” are mapped below. **(B)** Locations of selected tauopathy-associated mutations and phosphorylation sites are indicated. Orange: mutations linked to tau seeding activity. Gray: newly identified mutations with unknown seeding activity. Magenta: phosphorylation sites promoting tau aggregation. Lime green: phosphorylation sites inhibiting tau aggregation. **(C)** Distribution of other PTMs. Plum: ubiquitylation sites associated with enhanced seeding activity. Gray: ubiquitylation sites not affecting tau clearance. Mulberry: acetylation sites increasing tau aggregation. Forest green: dopamination. Amino acid positions are numbered relative to the tau441 isoform.

Under pathological conditions associated with AD, tau aggregates through nucleation-dependent polymerization (Vaquer-Alicea et al., 2021). Tau monomers shift conformation to form aggregation-competent intermediates, with the creation of the first oligomeric “nucleus” as the rate-limiting step (Vaquer-Alicea et al., 2021). Once seeded, tau rapidly forms β-sheet-rich fibrils (Dimou et al., 2023), which assemble into soluble oligomers and insoluble paired helical filaments (PHFs), eventually resulting in NFTs (Chu and Liu, 2019; Figure 2). Among these different species of aggregates, soluble high-molecular-weight aggregates (oligomers) characterized by a highly stable β-sheet core resistant to proteinase (Fitzpatrick et al., 2017), sodium dodecyl sulfate (SDS) and β-mercaptoethanol (Miao et al., 2019), along with post-translational modifications such as hyperphosphorylation and truncation (Zhou et al., 2018; Saroja et al., 2022), typically possess the most canonical prion-like properties–templating the misfolding and propagating pathology. These “pathological seeds” (Peng et al., 2020) vary in conformation, determining strain specificity. The following section reviews recent advances on tau’s intrinsic factors influencing seed properties.

**FIGURE 2:**
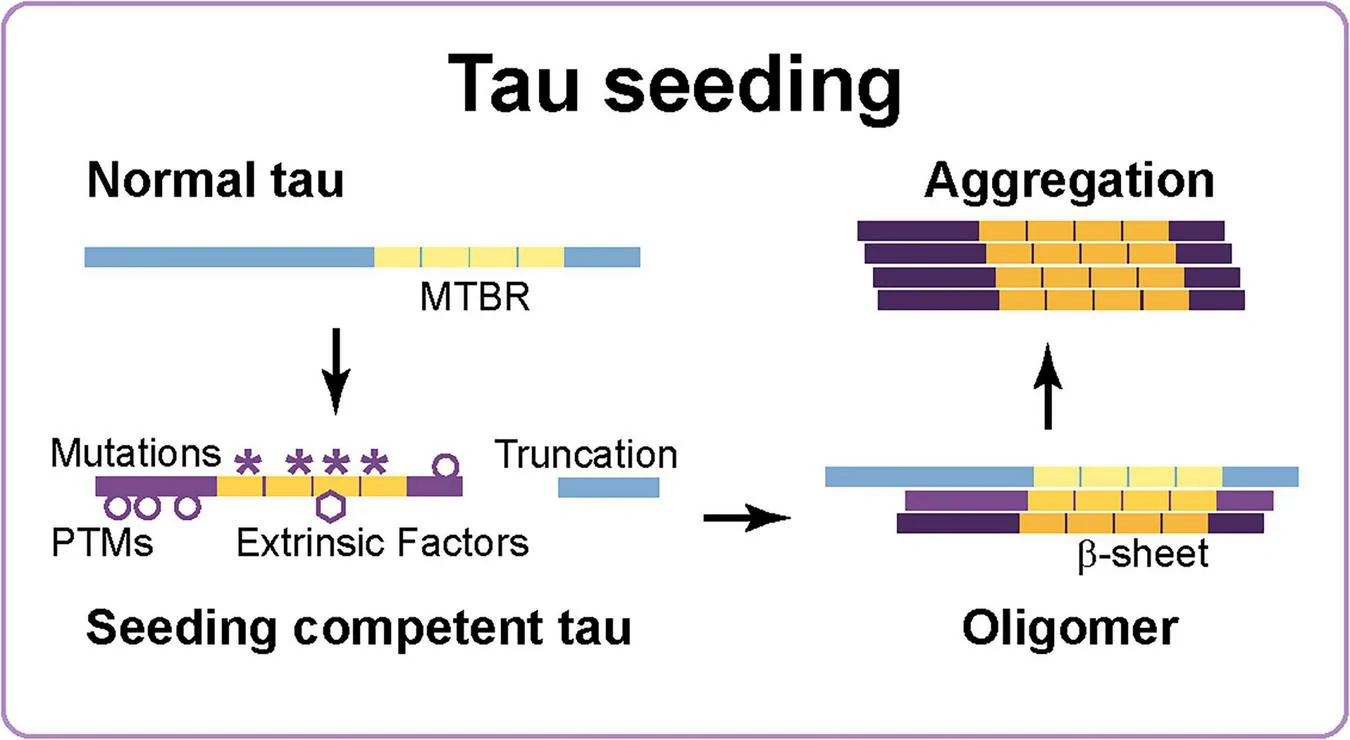
Schematic illustration of tau seeding and aggregation. Normal tau consists of four microtubule-binding regions (MTBRs) and flanking regions. Under pathological conditions, factors such as genetic mutations, post-translational modifications (PTMs), truncation, and extrinsic factors convert normal tau into a “seeding competent” state. These seeds then trigger the misfolding of normal tau proteins, promoting the formation of β-sheet-rich oligomers, which eventually assemble into large, insoluble aggregates. *, mutation.

### Conformational basis of tau seeds

Treatment of sarkosyl-insoluble fractions (tau seeds) extracted from patient brains with trypsin removes the N-terminal and C-terminal regions (the “fuzzy coat”) of the tau protein (Figure 1A). Notably, trypsinized tau seeds retain their ability to induce seeded aggregation like non-trypsinized seeds, indicating that the trypsin-resistant fibrillar core is the key determinant of tau’s prion-like properties (Vaquer-Alicea et al., 2021).

Several tau core sequences have been identified. For example, PHF43, which spans residues N265–E338 (ΔR2) in 0N3R tau (the smallest human tau isoform), exhibits an intrinsic ability to spontaneously aggregate (Von et al., 2000). Similarly, residues V306–F378 of the full-length 2N4R tau isoform also display spontaneous aggregation capacity (Carlomagno et al., 2021). Within these core sequences, hexapeptide interaction motifs play a pivotal role. The motif 306VQIVYK311 (PHF6), located at the beginning of the third MTBR, and its counterpart PHF6* (275VQIINK280 in R2), regulate tau assembly into fibrils via interactions with other regions of the sequence. These motifs serve as minimal interaction elements that initiate PHF assembly and promote β-structure formation (Von et al., 2000).

Recently, Louros et al. identified a novel amyloidogenic segment, PAM4 (Polymorphic Amyloid segment in repeat 4), comprising residues 350–362 of R4 (350VQSKIGSLDNITH362), and demonstrated that it self-assembles into amyloid fibrils. Cryo-EM analysis revealed that PAM4 fibrils are highly polymorphic. Remarkably, the polymorphs formed by this isolated segment recapitulate the conformations that PAM4 adopts in fibrils from various human tauopathies, including AD, corticobasal degeneration (CBD), and progressive supranuclear palsy (PSP). This study suggests that not only the “code” for tau aggregation, but also the determinants of tau seed polymorphism across different diseases, are embedded within the intrinsic tau sequence (Louros et al., 2024).

### Conformational polymorphism of tau strains

The tau protein can adopt multiple fibril polymorphs. In AD, for example, two distinct types of tau fibrils–PHFs and straight filaments–are observed. Both fibril types are assembled from two protofilaments through a C-shaped ordered core (G304–E380 in 2N4R tau), but with different packing arrangements (Todd et al., 2024). These tau folds have been identified in fibrils derived from sporadic and familial AD, Down syndrome-associated AD, familial British and Danish dementia, primary age-related tauopathy, prion-protein cerebral amyloid angiopathy (CAA), and Gerstmann–Sträussler–Scheinker disease (Scheres et al., 2023; Ghosh et al., 2024; Hoq et al., 2024).

In contrast, the structures of tau fibrils in other tauopathies—such as argyrophilic grain disease (AGD), CBD, chronic traumatic encephalopathy (CTE), globular glial tauopathy (GGT), Pick’s disease (PiD), and PSP—differ from those in AD, and the folds vary significantly across different diseases, enabling structure-based classification of tauopathies (Lovestam et al., 2022; Qi et al., 2023). The core regions of tau aggregates differ among diseases, underlying their conformational diversity (Chung et al., 2021). For instance, tau aggregates isolated from AD, PiD, and PSP represent distinct tau strains, each with unique ultrastructural properties and strain-dependent seeded tau aggregation (Tarutani et al., 2021; Scheres et al., 2023; Todd et al., 2024). Specifically, the PiD fold encompasses the core region K254–F378 (a more elongated fold including R1), while the CBD fold spans K274–E380 (covering the terminal residue of R1 and the entire R2 region). Both folds overlap with the AD fold core region, which consists of R3 and R4, highlighting the importance of these structural cores (Tarutani et al., 2021). Notably, similar to the AD core, the CBD and PiD cores can spontaneously self-assemble under physiological conditions without exogenous inducers (Carlomagno et al., 2021).

Seed-induced templated aggregation is the core mechanism underlying tau pathology propagation. Tau seeds derived from AD, PiD, and PSP retain their original ultrastructural and biochemical properties, as well as substrate selectivity, after amplification in cellular or animal models, indicating that specific conformational information can be stably transmitted across generations of propagation (Tarutani et al., 2021, 2023). Pathogenic tau seeds or extracellular vesicles isolated from the brain-derived fluid (BD-EVs) of various human tauopathies exhibit disease-specific seeding activities (Oosterlynck et al., 2026). The prion-like seeding activities follow this order: GGT-tau seeds > CBD-tau seeds > PSP-tau seeds > AD-tau seeds > PiD-tau seeds, a hierarchy that may reflect differences in the intrinsic properties of their conformational strains.(Chung et al., 2019; Tarutani et al., 2021). Similar to AD, specific tau species in certain tauopathies possess seeding activity; for instance, high-molecular-weight tau in PSP brains represents the principal form driving seeding activity (Martinez-Valbuena et al., 2025). In summary, tau conformational polymorphism determines the efficiency and substrate selectivity of templated propagation, serving as the molecular basis for the differential propagation patterns observed across various tauopathies.

Tau seed susceptibility is determined by both host-derived factors and strain-specific properties. First, host tau protein acts as an essential substrate for tau seed-induced pathology. For example, AD tau seeds do not induce pathology in tau-knockout mice, while mice expressing human tau are more susceptible to AD seeds than wild-type mice expressing only murine 4R-tau, indicating that the human tau sequence has a higher affinity for the AD conformation (Andrés-Benito et al., 2022). In human-mouse neuronal chimeric models, AD pathology is almost exclusively observed in transplanted human neurons, further confirming the species-specific susceptibility of human neurons to AD tau seeds (Ji et al., 2026). Second, the unique conformations of tau strains from different tauopathies appear to influence their preferred target cell types (Chung et al., 2021). AD tau seeds mainly target neurons, with hyperactive neurons—such as those in the hippocampal CA1 region and cortical COUP-TF-interacting protein 2 (CTIP2^+^) and somatostatin^+^ neurons—being particularly susceptible due to increased synaptic release and uptake (Weidling et al., 2025; Alexandersen et al., 2026). In contrast, PSP-tau and CBD-tau strains can induce tau inclusions in astroglia and oligodendroglia (Narasimhan et al., 2017). Moreover, tau mutations may also modulate the susceptibility of glial cells to tau seeds (Qi et al., 2026). Collectively, these findings highlight that the cell-type specificity of tau pathology is governed by both the intrinsic conformational properties of tau strains and the host cellular environment.

## Intrinsic factors influencing tau prion-like activity

Tau’s prion-like activity is governed by a variety of intrinsic factors, including genetic mutations, alternative splicing, post-translational modifications, and liquid–liquid phase separation (LLPS). Together, these elements shape tau’s conformational dynamics, aggregation propensity, and strain diversity.

### *MAPT* mutations

The *MAPT* gene contains over 100 coding mutations affecting more than 60 amino acid residues, in addition to 34 non-coding variants, the majority of which have been identified in patients with frontotemporal dementia (FTD)^1^. Well-established canonical missense mutations linked to FTD—such as P301L, P301S, R406W, V337M, ΔK280, and G272V—are known to promote tau fibrillization and are commonly utilized to model primary tauopathies in cellular and transgenic animal studies (Figure 1B; Limorenko and Lashuel, 2022). Furthermore, mutations within the introns of the *MAPT* gene that disrupt the physiological balance of tau alternative splicing also affect tau aggregation (Parra Bravo et al., 2024) (see section Alternative splicing).

#### P301L and P301S

P301L and P301S (in the R2 MTBR) are the most common MAPT mutations linked to familial FTDP-17, making them key models for tau aggregation, strain diversity, and prion-like spread. Cryo-EM of P301L patient brains revealed a unique three-lobed tau fold similar to PiD and distinct from wild-type or other mutations (Schweighauser et al., 2025). Complementing these structural studies, all-atom replica-exchange molecular dynamics (REMD) simulations on the tau jR2R3 fragment (residues 295–313, spanning the R2/R3 junction) containing the P301L mutation further demonstrated that P301L preorganizes tau into an aggregation-prone conformation (Longhini et al., 2024), efficiently templating 4R-tau but not 3R-tau. These atomic-level findings provide a mechanistic explanation for the robust prion-like propagation of P301L tau: its unique folded architecture serves as an efficient conformational template, triggering structural conversion and pathological aggregation of endogenous 4R tau.

Biophysical studies using single-molecule Förster resonance energy transfer (FRET), nuclear magnetic resonance (NMR), and electron spin resonance (ESR) (Sinha Roy et al., 2026) demonstrated that both mutations promote extended tau conformations of the N-terminal and C-terminal regions, increase MTBR exposure (Wen et al., 2021; Stelzl et al., 2022), and lower aggregation barriers, with solvent effects further facilitating β-sheet stacking (Vigers et al., 2025).

Complementing these biophysical insights, histopathological studies have delineated the regional distribution of tau pathology in presymptomatic and early-stage *MAPT* mutation carriers. In a study by Giannini et al. (2023), a presymptomatic P301L carrier exhibited the highest tau burden in the anterior cingulate, anterior temporal, middle/superior frontal, fronto-insular cortex, and amygdala. In contrast, a presymptomatic L315R carrier demonstrated limited tau pathology in the middle frontal, antero-inferior temporal, amygdala, hippocampus, and striatum. Meanwhile, an early-stage G272V carrier displayed predominant tau accumulation in the superior frontal and anterior cingulate cortices, as well as in the subiculum and CA1 region. These findings reveal that *MAPT* mutations exhibit distinct early-stage distribution patterns, highlighting mutation-specific topographic heterogeneity in the initiation and early spread of tau pathology.

Collectively, research on the P301 tau mutations has progressed from a purely phenomenological description of pathology to atomic-level structural characterization and quantitative insights into the dynamic conformational changes that underlie tau seeding propensity and prion-like propagation. However, despite P301L and P301S being cornerstone models for tau seeding and pathogenesis, significant structural discrepancies exist between filaments derived from human brains and those generated in widely used transgenic mouse lines. Generally, tau filament cores from rTg4510, Tg2541, and PS19 mice are shorter than those purified from human tauopathy brains (Zhang et al., 2020; Scheres et al., 2023; Schweighauser et al., 2023; Zhao et al., 2024), casting doubt on whether mutation-driven seeding mechanisms gleaned from these models are applicable to sporadic human tauopathies. Another major caveat is that most *in vivo* mechanistic studies depend on transgenic overexpression of mutant tau, rather than physiological expression of endogenous mutants. This approach risks artificially amplifying aggregation and may mask the modulatory role of wild-type tau under heterozygous conditions. Consequently, current transgenic models expressing P301 mutants should be interpreted with caution when studying AD or sporadic 4-repeat tauopathies. The challenge of creating mouse models that faithfully replicate human tau filament folds remains an open issue.

#### R406W and V307M

Beyond P301L and P301S, additional MAPT mutations–including R406W, V337M, G272V and ΔK280–have been shown to modulate tau seeding and prion-like propagation through distinct mechanisms. Cryo-EM structural analyses reveal that both V337M and R406W mutations adopt the “Alzheimer fold” in patient brains, with all cases of V337M and R406W exhibiting PHFs. Moreover, the V337M mutation significantly accelerates tau aggregation kinetics *in vitro* (Qi et al., 2025). Mechanistically, these two mutations exhibit both convergent and divergent downstream pathways. V337M promotes tau prion-like seeding through multiple mechanisms, including the activation of stress granule formation, impairment of autophagic function, and disruption of astrocytic cholesterol homeostasis (Bowles et al., 2021; Glasauer et al., 2022). In contrast, R406W disrupts autophagic flux either by directly affecting the autophagy-lysosome pathway (ALP) (Mirfakhar et al., 2026) or by upregulating GRAM Domain Containing 1B (G RAMD1B) (Acosta Ingram et al., 2025), which leads to the lysosomal retention of phosphorylated tau and subsequently accelerates tau aggregation and spreading.

#### ΔK280

The ΔK280 mutation, characterized by a deletion of lysine at position 280 within the MTBRs of tau, is recognized for its role in accelerating fibril formation in both full-length tau (Chen G. J. et al., 2025) and the truncated K18 construct (Chen Y. et al., 2025) that includes the MTBRs. *In vitro* studies show that while wild-type full-length tau predominantly forms trimeric species, the ΔK280 variant tends to assemble into larger aggregates and demonstrates enhanced cell-to-cell transmission. Notably, cross-seeding experiments indicate that although wild-type tau is more efficiently seeded by its own fibrils compared to those from the ΔK280 mutant, the latter can effectively convert wild-type tau into fibrils that display structural characteristics akin to the mutant (Chen G. J. et al., 2025). Additionally, all-atom molecular dynamics simulations have shed light on the intrinsic properties of the ΔK280 mutation, revealing that it significantly increases the degree of collapse and promotes β-sheet formation among six specific hexapeptide motifs: ^264^ENLKHQ^269^, ^275^VQIINK^280^ (designated PHF6*), ^295^DNIKHV^300^, ^306^VQIVYK^311^ (PHF6), ^326^GNIHHK^331^, and ^337^VEVKSE^342^. This mutation enhances the hydrophobic solvent-accessible surface area (SASA) of these critical motifs and strengthens their hydrophobic interactions, hydrogen bonds, and cation-π interactions, thereby facilitating LLPS of the K18 fragment (Chen Y. et al., 2025). These insights contribute to a deeper structural and mechanistic understanding of the aggregation and propagation processes driven by the ΔK280 mutation in tau.

#### Emerging MAPT mutations

Beyond the well-established mutations, recent genetic screening efforts—particularly in non-Caucasian populations–have identified novel MAPT variants. In a Chinese cohort of FTD patients, exome sequencing revealed multiple novel MAPT mutations, including D54N, E342K, R221P, and T263I (Nan et al., 2024). Another Chinese FTD cohort identified the mutations MAPT R406Q, D430H, and A330D (Cheng et al., 2023). Functional analyses demonstrated that cells expressing R406Q and D430H tau exhibited higher levels of phosphorylated tau compared to those expressing wild-type tau, particularly at the Thr205, Thr231, and Ser396 phosphorylation sites. Furthermore, these variants impaired tau’s ability to bind to microtubules and increased tau self-aggregation (Cheng et al., 2023).

In patients with progressive supranuclear palsy-Richardson’s syndrome (PSP-RS), the R406Q mutation was also reported (Badihian et al., 2024), along with E342K (Li et al., 2024). The E342K mutation was associated with atypical features resembling α-synucleinopathies, such as autonomic dysfunction and dream enactment behavior. Both the R406Q and E342K mutations were linked to AD-like characteristics, including poorer performance on memory tests (Badihian et al., 2024). Additionally, analysis of a whole-exome sequencing database identified a novel I308T variant in a patient with early-onset amyotrophic lateral sclerosis (ALS). In motor neuron cells expressing I308T mutants, tau distribution was found to be discontinuous along neurites, accompanied by mitochondrial clustering due to impaired mitochondrial motility (De Bertier et al., 2025).

In summary, among these emerging MAPT mutations, R406Q and D430H have been shown to promote tau phosphorylation and self-aggregation at the cellular level, directly implicating them in enhancing tau seeding propensity. However, the precise effects of other mutations–including E342K, I308T, D54N, R221P, T263I, and A330D–on tau seeding and prion-like activity require further investigation.

#### Chromosomal variations

In addition to point mutations in the *MAPT* gene, chromosomal variations in the 17q21.31 region significantly contribute to the risk of tauopathies. *In vitro* seeding experiments indicated that brain homogenates from individuals carrying *MAPT* gene microduplications (which involve microduplications of the 17q21.31 chromosomal region encompassing the MAPT gene) were capable of inducing tau aggregates in Human Embryonic Kidney (HEK)-biosensor cells. These aggregates exhibited morphological features that were intermediate between those generated by homogenates from cases of PiD and PSP (Wallon et al., 2021). This suggests that such duplications can facilitate tau aggregate formation and generate mixed tau conformational strains. Moreover, specific *MAPT* haplotypes, which are known to influence gene expression and alternative splicing, may heighten the risk of sporadic FTD (Manzoni et al., 2024) and are associated with distinct patterns of tau pathology spread across regions (Cullinane et al., 2026). Collectively, these findings imply that *MAPT*-related chromosomal variations might modulate tau seeding activity and the efficiency of transregional propagation, primarily by altering levels of tau expression, isoform ratios, and conformational attributes. This modulation could exacerbate the burden of tau pathology and contribute to the heterogeneity observed in these diseases.

### Alternative splicing

Alternative splicing of *MAPT* exon 10 produces 3R- and 4R-tau isoforms (Goedert et al., 1989). Under physiological conditions, 4R-tau exhibits greater microtubule-binding affinity and polymerization efficiency than 3R-tau. In the adult human brain, all six tau isoforms are expressed in roughly equal 3R:4R ratios, whereas adult rodents mainly express 4R-tau. Tauopathies disrupt this balance: PSP and CBD are over 90% 4R-tau, PiD is predominantly 3R-tau, and AD, CTE, and primary age-related tauopathy (PART) have mixed 3R/4R aggregates, with ratios varying during disease progression (Parra Bravo et al., 2024). These isoform differences affect tau aggregation and seeding activity.

MAPT intronic mutations disrupt exon 10 splicing, leading to 3R/4R imbalance and altered tau pathology (Maxwell et al., 2021; Olszewska et al., 2021; Mori et al., 2023). More than a dozen such intronic mutations have been identified in *MAPT* intron 9 or intron 10, and these can either promote or suppress exon 10 inclusion. The extensively studied FTD-linked IVS10+16 mutation, for example, increases 4R-tau, leading to increased cortical tau accumulation and neuronal dysfunction (Dando et al., 2024; Cordella et al., 2025). Thus, altered tau splicing critically drives aggregation.

Tau seeds from diseased brains show remarkable isoform selectivity (Chung et al., 2021): AD seeds incorporate both 3R- and 4R-tau (all six isoforms) (Wu et al., 2022), CBD seeds only 4R, and PiD seeds only 3R, mirroring disease-specific pathology (Hosokawa et al., 2022; Duan et al., 2024; Gabani et al., 2026). This seeding specificity is maintained through cellular amplification across multiple passages (Tarutani et al., 2021; Gabani et al., 2026). Huntington’s disease seeds do not induce aggregation of either 3R- or 4R-tau (Tarutani et al., 2021). Together, these findings demonstrate that tau seeds from different tauopathies exhibit isoform-specific recruitment patterns, and importantly, this seeding specificity is faithfully maintained upon cellular amplification.

The prion-like activity of 3R- and 4R-tau is debated. *In vitro*, 3R tau aggregates faster than 4R tau under both unseeded conditions and when seeded with AD brain homogenates (Wu et al., 2022). However, the 19-residue 4R-derived peptide jR2R3 can fibrillize and be seeded, while the 3R-derived jR1R3 peptide (spanning the R1–R3 junction) does not fibrillize spontaneously, in the presence of heparin, or upon seeding (Longhini et al., 2024). The authors propose that the differential prion-like activities between 4R- and 3R-derived peptides may result from variations in the N-terminal-flanking residues adjacent to the PHF6 motif—specifically, the amino acid at position 305 (serine in 4R versus lysine in 3R)—which modulates seeding efficiency (Longhini et al., 2024). Nevertheless, AD tau filaments recruit both isoforms without significant difference in cells (Gabani et al., 2026), suggesting experimental context and fragment selection are critical.

Traditional models propose an ‘asymmetric barrier’ in cross-seeding, where 4R tau fibrils are ineffective at recruiting 3R tau monomers (Dinkel et al., 2011; Siddiqua et al., 2012; Longhini et al., 2024). In human iPSC-derived neurons, co-expression of 3R and 4R tau reduces pathology relative to 4R alone, supporting that 4R tau preferentially aggregates with itself (Dannert et al., 2026). Nevertheless, cross-seeding between isoforms can occur under certain conditions. For example, in a rhesus monkey model, AAV-delivered human 4R tau (carrying P301L/S320F) not only propagated itself but also recruited endogenous monkey 3R tau via a ‘permissive templating’ mechanism, suggesting that 4R tau may have cross-isoform seeding capacity (Beckman et al., 2021). By contrast, PiD-derived 3R tau seeds fail to recruit 4R tau, indicating that cross-seeding may be unidirectional or dependent on specific conformational states. The molecular basis for this selective recruitment lies in the conformational strain properties of the seed fibrils—specifically, whether tau monomers adopt an extended or compact conformation determines their ability to be templated by pathological fibrils (Dannert et al., 2026).

Although 3R- and 4R-tau isoforms differ in aggregation and seeding selectivity, conclusions regarding their differential seeding activities should be interpreted with caution, taking into account the specific experimental systems employed. First, discrepancies could arise from differences in reaction systems. Most *in vitro* aggregation assays rely on polyanionic inducers such as heparin, which may bypass or alter the aggregation pathways mediated by endogenous mechanisms—including phosphorylation and LLPS–under physiological conditions in vivo, thereby potentially biasing the assessment of the intrinsic seeding competence of specific isoforms. On the other hand, cell-based assays, while more physiologically relevant, typically operate within a time window of days to weeks, which is insufficient to recapitulate the chronic, years-to-decades-long progression of human tauopathies. Second, the tau fragments used across different studies vary considerably in length. Truncation may expose or conceal key conformational regulatory elements, leading to contradictory conclusions regarding isoform selectivity derived from different truncated constructs. Therefore, the molecular basis of 3R/4R seeding selectivity warrants further validation in models that more faithfully recapitulate human diseases.

### Post-translational modifications shaping seed activity

Post-translational modifications (PTMs) of tau–including phosphorylation, truncation, glycosylation, ubiquitylation, acetylation, and dopamination–play a pivotal role in the acquisition and maintenance of prion-like seeding activity (Kumar et al., 2024). While sarkosyl-insoluble extracts from AD brains exhibit limited capacity to recruit and polymerize additional monomers after inducing full-length tau aggregation *in vitro* (Duan et al., 2024), tau seeds amplified in cells retain both the prion-like seeding activity and isoform specificity of the original seeds (Tarutani et al., 2021). This discrepancy underscores the essential role of intracellular PTMs in generating functional seeding activity.

#### Phosphorylation

Disease-specific hyperphosphorylation is among the most critical PTMs that characterize AD tau. AD samples display markedly higher levels of AT8-, pT217-, and pT181-positive aggregates versus other tauopathies and controls. In AD, approximately 54% of AT8-positive aggregates are co-labeled with pT181 and 68% with pT217, compared to less than 20% in PSP, CBD, and PiD, and under 10% in healthy controls (Böken et al., 2026). Conversely, PSP tau exhibits preferential phosphorylation at Ser356, with pS356-positive aggregates most abundant in PSP and nearly absent in AD (Böken et al., 2026).

Hyperphosphorylation is a critical post-translational modification that shifts tau from its normal, reversible state to pathological, irreversible aggregation (Figure 1B). Physiological phosphorylation regulates tau folding, but disease-related hyperphosphorylation causes tau to dissociate from microtubules and assemble into β-sheet amyloid fibrils (Masquelier et al., 2026). Hyperphosphorylated tau monomers can induce neurotoxicity independently of fibril formation, leading to neurite degeneration, synaptic loss, and behavioral deficits (Watamura et al., 2025). In AD model animals, regions with the highest tau prion-like activity (the limbic corticostriatal system) also show the most pronounced phosphorylated tau accumulation (AT8-positive) (Ayers et al., 2024). Clinically, tau seeding activity in cerebrospinal fluid strongly correlates with total and phosphorylated tau levels (e.g., pTau212/214, pTau181), and increases with Braak stage, establishing a direct link between phosphorylated tau, prion-like activity, and neurodegeneration (Jin et al., 2022; Frey et al., 2023; Houzé et al., 2025; Weber et al., 2026).

Recent studies show that phosphorylation at specific sites in the “fuzzy coat” regions flanking the tau filament core (a.a. 1–305, 379–441) is essential for full seeding activity. The PAD12 tau mutant, with twelve phospho-mimetic residues (T181D, S202D, S205D, T212D, S214D, T217D, T235D, T231D, S396D, S400D, T403D, and S404D), mimics the core structure of AD tau and imparts full seeding competence to recombinant tau (Kasen et al., 2025; Lövestam et al., 2026). These phosphorylation patterns are replicated during templated fibril formation (Tarutani et al., 2023). Beyond neuronal cells, microglia can also phosphorylate tau (pS184, pS198) after internalizing fibrils, and this tau can efficiently seed aggregation in neurons (Karabova et al., 2026). Thus, fuzzy coat phosphorylation is critical for replicable, transgenerational tau seeding.

Phosphorylation within the MTBR region also modulates tau seeding activity in a site-specific manner. Fyn phosphorylates tau at Y310, which recruits GSK3β to further phosphorylate tau, granting full seeding capacity (Li et al., 2025). Intriguingly, phosphorylation at S324 or S356 inhibits oligomerization and seeding of the V306–F378 fragment (Le et al., 2023), and phospho-mimetic mutations at S262/T263/S289/S305–especially S305–reduce AD tau seeding efficiency (Smith et al., 2023). These observations highlight the nuanced, site-specific regulation of tau seeding by phosphorylation.

Given its central role as both a molecular switch for prion-like activity and a disease-defining signature, phosphorylation represents a promising target for therapeutic intervention. Thus, beyond its diagnostic value as a core biomarker (Wu et al., 2025), targeting tau phosphorylation may offer a strategy to block the templated spread of tau pathology (Su et al., 2024; Xiao et al., 2024; Yao et al., 2024).

#### Truncation

Analogous to phosphorylation, tau truncation is a vital PTM that alters tau’s conformational stability and potently modulates its prion-like seeding competence. Over 40 distinct truncated tau species have been identified in AD brains and models (Chu et al., 2023).

Truncated forms retaining the MTBR and flanking regions—especially with N- or C-terminal deletions–show increased aggregation, correlate with disease, and can seed full-length tau (Miyoshi et al., 2021; Chu et al., 2023). For example, while *in vivo* propagation is unproven, the tau_297–391_ fragment forms fibrils *in vitro* that resemble those from AD and CTE brains (Lovestam et al., 2022), whereas unphosphorylated tau_151–391_ does not (Lovestam et al., 2022). Notably, our lab generated 12 tau isoforms truncated at 51, 151, 231 (N-terminal) and 391, 421 (C-terminal), plus full-length tau. Of these, tau_151–391_ exhibits the strongest pathological activities in cultured cells, including promoting tau phosphorylation, self-aggregation, and seeded aggregation by AD-derived pathological tau (AD P-Tau) (Gu et al., 2020; Jin et al., 2022). Thus, truncated tau likely requires further modification (e.g., phosphorylation) within the cellular environment to form fully pathogenic fibrils.

Truncation not only promotes tau aggregation but is also reciprocally facilitated by mature aggregates, which drive further tau cleavage. In human neuroblastoma SH-SY5Y cells, templated fibril formation induces tau truncation at both termini, suggesting truncation could happen during fuzzy-coat degradation after the fibril core forms (Tarutani et al., 2023).

Truncated tau lacking an intact MTBR has distinct properties. Xu and colleagues demonstrated that transcription factor EB (TFEB)—a master regulator of lysosomal biogenesis—drives lysosomal exocytosis that preferentially secretes MTBR-deficient truncated tau. Importantly, these secreted fragments lack pathological seeding capacity (Xu et al., 2021).

Furthermore, truncated tau-derived seeds contribute to the conformational diversity of nascent progeny fibrils. Zhang et al. used mutant tau_297–407_ (4E-tau) seeds, containing four phosphomimetic mutations (S396E, S400E, T403E, S404E), to accelerate full-length tau fibrillization. However, the resulting fibril core didn’t match the original seed’s structure. They proposed that truncated seeds promote fibril heterogeneity via secondary nucleation: full-length tau binds variably to seed surfaces, producing polymorphic fibrils (Zhang et al., 2026).

In summary, truncation’s effect on tau seeding depends on the MTBR, with bidirectional crosstalk between fragments and aggregates. Future studies are needed to dissect the molecular interplay between truncation and other PTMs (such as phosphorylation) and their synergistic regulation of pathological tau seeding.

#### Ubiquitylation

Dysfunction of the ubiquitin-proteasome system (UPS) is common in neurodegenerative diseases, and aberrant tau ubiquitination is closely associated with the seeding activity of tau aggregates. Mass spectrometry and *in vitro* assays show that both aqueous and sarkosyl-insoluble tau from AD brains have PTMs like ubiquitination, phosphorylation, and acetylation (Kumar et al., 2024). In AD brains, site-specific ubiquitin chain linkages on tau determine aggregate fate. Tau oligomers exhibit significantly higher K63-linked ubiquitination than K48-linked forms, and K63-linked oligomers are strongly associated with enhanced seeding activity and pathology. Tau seeds can promote intracellular K63-linked oligomer formation and secretion, suggesting that site-specific ubiquitination may signal enhanced tau seeding (Figure 1C; Puangmalai et al., 2022).

Ubiquitinated tau can be degraded by the UPS (Wang L. et al., 2025). CHIP, the main tau E3 ligase, recognizes phosphorylated tau through its tetratricopeptide repeat (TPR) domains and rapidly ubiquitinates it, but not unmodified tau. Even low levels of CHIP suppress phosphorylated tau aggregation and seeding (Nadel et al., 2023). CHIP also ubiquitinates K18 filaments at residues (K267, K274, K281, K290, K311, K353, and K369/370) (Parolini et al., 2023) overlapping with those found in tau PHFs from AD brains (Wesseling et al., 2020). However, ubiquitination minimally affects the tau fibril core (Parolini et al., 2023), suggesting that many ubiquitin modifications occur on accessible, non-core regions and don’t disrupt the core structure, potentially explaining why PHFs resist ubiquitin-mediated clearance.

Moreover, ubiquitinated tau aggregates can also be cleared by autophagy. A genome-wide screen recently identified a novel microautophagy-dependent pathway where the Endosomal Sorting Complexes Required for Transport-I (ESCRT-I) subunit Tumor Susceptibility Gene 101 (TSG101) recognizes polyubiquitinated tau aggregates, and the accessory protein Protein Tyrosine Phosphatase, Non-Receptor Type 23 (PTPN23) bridges ESCRT-I and ESCRT-III to facilitate engulfment and clearance. These findings illustrate the diversity of clearance mechanisms for ubiquitinated tau aggregates (Hirayama and Murata, 2025).

In a related context, mature tau fibrils exhibit significant resistance to proteasomal degradation due to the frequent absence of ubiquitination sites within their fibril core. The UPS is primarily responsible for clearing tau monomers and small oligomers, but proves largely ineffective at degrading mature fibrils. Alternatively, the ALP serves as a crucial mechanism for the removal of tau aggregates and pathological seeds, a process tightly regulated by the master transcription factors TFEB and transcription factor binding to IGHM enhancer 3 (TFE3) (Zhu et al., 2023; Zheng et al., 2025). Harnessing these intrinsic cellular degradation pathways to develop novel targeted protein degradation strategies has emerged as a promising direction for AD therapy, offering a powerful approach to selectively eliminate pathogenic tau seeds while circumventing the limitations associated with conventional enzyme inhibition (see Other Therapies).

### Acetylation

Acetylation of tau protein regulates its aggregation and seeding by neutralizing lysine residues, which relieves their inhibitory effect on aggregation and stabilizes the amyloid conformation (Li et al., 2023b). Site-specific acetylation (Figure 1C), such as at K280 within the VQIINK motif, enhances tau secretion, aggregation, and seeding (Song et al., 2023). Acetylation at K274, K311, and K369 increases tau fragment aggregation (Tseng et al., 2021; Li et al., 2023b), while simultaneous acetylation of K267, K274, and K280 markedly boosts R1R2 peptide seeding (Li et al., 2023b). Thus, acetylation is sufficient to confer seeding activity to tau.

Conversely, neutralizing acetylated tau with specific antibodies (see section 4) (Song et al., 2023) or genetically reducing site-specific tau acetylation ameliorates pathological aggregation (Pan et al., 2026). Mechanistically, UNC-51-like autophagy activating kinase 1 (ULK1) enhances autophagy and mitophagy, resulting in increased NAD^+^ and activating the NAD+-sirtuin 1 axis, which deacetylates acetylated K174 and reduces tau pathology (Pan et al., 2026).

#### Dopamination

Dopamine-induced tau modification (dopamination) is a recently identified PTM that has been implicated in mitigating tau pathology (Wang Q. et al., 2025). Dopamine modification at C322 of tau suppresses phosphorylation at several pathological sites (Figure 1C), including pS262, pS352, and pS324. Moreover, dopaminated tau exhibits greatly diminished fibrillization *in vitro* and reduced seeding activity in cellular models. Cryo-EM structural analysis of dopaminated tau fibrils reveals an exceptionally small fibril core comprising only 11 residues, including the critical VQIVYK motif—representing the smallest core reported for any tau fibril to date. This minimal core forms a limited hydrophobic interface, providing a structural explanation for the markedly reduced stability of dopaminated fibrils (Liu et al., 2026).

Taken together, PTMs exert profound effects on tau seeding activity. Notably, different PTMs—such as phosphorylation, acetylation, and ubiquitination—do not occur independently but are subject to complex crosstalk. Future research should systematically dissect how the PTM codes influences tau conformation and seeding activity, and developing therapeutic strategies targeting specific PTM networks.

### Liquid-liquid phase separation (LLPS)

As an intrinsically disordered protein, tau possesses a remarkable capacity for LLPS, conferred by its primary sequence and structural features. The liquid condensates formed by tau via LLPS act as “precursors” for the subsequent development of solid amyloid fibrils (Boyko and Surewicz, 2022). Mechanistically, LLPS induces local concentration of tau and promotes conformational rearrangements—shifting tau from a compact state to an extended conformation with exposed N- and C-termini, thereby unveiling the microtubule-binding region. These changes facilitate intermolecular interactions and ultimately accelerate fibril formation (Wen et al., 2021; Wen et al., 2025).

Disease-associated mutations, such as P301L/S, further accelerate this process (Wen et al., 2021; Martínez-Mármol et al., 2023). PTMs of tau–including phosphorylation (Powell et al., 2024; Allahyartorkaman et al., 2025), acetylation (Powell et al., 2024), and glycosylation (Powell et al., 2025)–have also been shown to regulate tau LLPS and subsequent aggregation. Nevertheless, results remain inconsistent across different *in vitro* experimental conditions.

Beyond tau’s intrinsic properties, various extrinsic molecules influence tau aggregation by modulating LLPS. For instance, RNA acts as a multivalent anionic scaffold that participates in and regulates tau LLPS (Allahyartorkaman et al., 2025). Certain scaffold proteins, such as the autophagy adaptor TRIAD3A, can form droplets through their own LLPS, recruiting and concentrating tau within these condensates (Zhou et al., 2024). Additionally, other pathological proteins–including Transactive response DNA-binding protein 43 (TDP-43) and Aβ40–enhance tau LLPS, thereby promoting amyloid fibril formation and cytotoxicity (Monnaka et al., 2026; Sun et al., 2026). In summary, tau LLPS is governed by both intrinsic factors and extrinsic regulators. The convergence of these factors on LLPS highlights its central role as a mechanistic hub linking tau’s molecular properties to its pathological aggregation and seeding activity.

## Extrinsic factors regulating tau prion-like activity

Tau aggregation and seeding activity are regulated by various extrinsic factors, including cofactors (such as polyanions), environmental conditions (like pH, high salt, and molecular crowding), molecular chaperones, and protein homeostasis mechanisms (Vaquer-Alicea et al., 2021). In recent years, research extending beyond intrinsic regulatory mechanisms has significantly enhanced our understanding of how extrinsic factors modulate tau seeding.

### Cofactors

The MTBR of tau is characterized by a high density of positive charges (Chu et al., 2023). Various polyanionic substances–including heparin, RNA, and lipids–can interact with these positively charged regions of tau through electrostatic interactions. This binding neutralizes intramolecular repulsive forces within tau, induces conformational changes, and promotes tau aggregation (Vaquer-Alicea et al., 2021). Beyond these established polyanions, recent studies have demonstrated that polyserine expression further exacerbates tau phosphorylation and enhances its seeding capacity through a similar mechanism (Van Alstyne et al., 2025, 2026).

### Molecular chaperones

Advances in tau seeding research have revealed that various molecular chaperones *in vivo* directly bind tau and regulate its aggregation and seeding. The classical heat shock protein (Hsp) family, especially Hsp70/Hsc70 and Hsp90, forms a multichaperone complex that binds the tau repeat region and blocks aggregation (Saha et al., 2023; Di Lorenzo et al., 2025). Hsp27 (HSPB1) specifically recognizes and binds phosphorylated tau sites, inhibiting fibril formation (Yang et al., 2024). Hsp40 family members (DNAJA2, DNAJB1, DnaJC7) bind tau monomers, seeds, or fibrils, suppressing aggregation and modulating seeding (Hou et al., 2021; Irwin et al., 2021).

Extracellular chaperones also contribute to tau seeding. Clusterin is a 70 kDa chaperone-like glycoprotein that is predominantly present in plasma and extracellular fluid, and its levels are elevated in AD patients. Typically, Clusterin serves to stabilize unfolded proteins, preventing their aggregation, and acts to inhibit the fibril formation of Aβ, α-synuclein (α-Syn), and other amyloidogenic proteins *in vitro*. However, in relation to tau pathology, Clusterin binds to and stabilizes soluble oligomeric tau species, keeping them in a highly seeding-competent state. These Clusterin/tau complexes can then enter recipient cells through HSPG-dependent endocytosis. Once inside, these composite seeds trigger vesicle rupture and escape from endosomes into the cytosol, which facilitates the templated aggregation of endogenous tau (Yuste-Checa et al., 2021).

Numerous other chaperones have been reported to regulate tau aggregation, including Tripartite motif containing 11 (TRIM11) (Zhang et al., 2023), S100 calcium-binding protein B (S100B) (Moreira et al., 2021), FK506-binding protein 12 (FKBP12) (Jiang et al., 2023), FK506-binding protein 51 (FKBP51) (Chakraborty and Zweckstetter, 2025), BAG cochaperone 3 (BAG3) (Augur et al., 2025), Prefoldin 5 (Bisht et al., 2026), chaperonin containing TCP1 subunit 2 (CCT2) (Ma et al., 2022), VCP (Saha et al., 2023), Hsp90 co-chaperone p23 (Moll et al., 2022), CHIP (Nadel et al., 2023), among others. Notably, most of these chaperones inhibit tau aggregation.

These insights support developing targeted chaperones [e.g., Bri2 BRICHOS (Su et al., 2026)] or synthetic peptides like β-Hsp90, a β-hairpin mimetic derived from the Hsp90–tau interaction sequence (Di Lorenzo et al., 2025), which inhibit tau aggregation, as potential therapies for tauopathies.

### Non-coding RNAs and epigenetics

Genetic polymorphisms like those in Apolipoprotein E (ApoE) contribute to tau pathology (Fernández-Calle et al., 2022), however, recent research has uncovered critical roles for non-coding RNAs and epigenetic mechanisms in regulating tau seeding. In the temporal superior T1 isocortex of AD brains, miR-146b-5p and miR-151a-5p levels correlate with Braak stage and tau phosphorylation; miR-146b-5p mimics enhance, while miR-151a-5p mimics reduce tau seeding in biosensor assays. These miRNAs likely regulate tau phosphorylation by targeting kinases such as GSK3β and cyclin dependent kinase 5 regulatory subunit 1 (CDK5R1) (Nagaraj et al., 2026).

Long non-coding RNAs (lncRNAs) also affect tau. The lncRNAs SNHG8 is reduced in mouse models of tauopathy and in the brains of patients with FTLD, PSP, and AD. SNHG8 inhibits stress granule formation by binding both tau and TIA1 Cytotoxic Granule Associated RNA Binding Protein (TIA1), a core stress granule protein (Bhagat et al., 2023). As stress granules are critical for tau seeding and prion-like propagation (Younas and Zerr, 2025), the loss of SNHG8 is predicted to enhance stress granule-mediated tau aggregation and spread.

Epigenetic regulation further influences tau seeding. ACSS2 (acetyl-CoA synthetase 2), a nuclear metabolic enzyme, maintains histone acetylation and gene expression for learning. Loss of ACSS2 in hippocampal pyramidal neurons and Cajal-Retzius cells exacerbates tau seeding-related phenotypes, implicating the metabolic-epigenetic axis in tau seeding regulation (Egervari et al., 2026). However, studies on the epigenetic regulation of tau seeding remain largely at the level of phenotypic correlation, and direct evidence demonstrating the causal effects of epigenetic regulators on tau conformation and seeding efficiency is still lacking.

### Other factors

Various environmental exposures, such as PM2.5 (Liu et al., 2024), chronic intermittent hypoxia (Kazim et al., 2022), and traumatic brain injury (Zhao et al., 2023; Kimura et al., 2026), have been shown to exacerbate tau seeding and its transregional propagation. Additionally, metal ions (like copper and zinc) have been reported to promote tau aggregation (Gao et al., 2022). Beyond these physical and chemical environmental influences, the immune microenvironment, particularly in response to viral infection, also modulates tau prion-like activity. For example, type I interferon (IFN-I), induced by the viral nucleic acid mimic PolyI:C, has been found to enhance tau aggregation, while genetic ablation of the Type I Interferons receptor Ifnar1 attenuates tau pathology (Sanford et al., 2024).

Beyond the intrinsic and extrinsic factors directly influencing tau prion-like activity discussed above, tau seeds also spread through intercellular transmission. Following secretion (Xing et al., 2026), they are affected by extracellular factors and interactions with various cell types (Olesen et al., 2025), then internalized by connected neurons (Li et al., 2026), thereby driving propagation (Parton et al., 2026).

### Trans-seeding

Flach et al. demonstrated that fibrils of the yeast prion domain Sup35NM can act as heterologous seeds, promoting the formation of morphologically distinct tau fibril strains *in vitro*. Intrahippocampal injection of Sup35NM fibrils into P301S tau transgenic mice exacerbated tau pathology. These findings provide the first *in vivo* evidence for heterotypic cross-species seeding of a neurodegenerative human prion-like protein by a yeast prion–a mechanism termed “trans-seeding” (Flach et al., 2022).

Subsequent studies have identified several additional proteins–including α-Syn (Zhan et al., 2025), islet amyloid polypeptide (IAPP) (Zhang et al., 2022), the yeast protein Ure2p from Saccharomyces cerevisiae (Meng et al., 2023), the cofilin (1–138) fragment generated by Asparagine Endopeptidase cleavage of the actin-binding protein cofilin (Yan et al., 2023), and the bridging integrator 1 (BIN1) (1–277) fragment produced by legumain cleavage of BIN1 (Zhang X. et al., 2024)–that can interact with tau and co-aggregate to form more toxic, trans-seeded tau fibrils. These seeds display enhanced seeding activity and neurotoxicity compared to pure tau fibrils *in vitro*, and they further exacerbate tau pathology and cognitive deficits in AD mouse models. Collectively, these observations provide a potential mechanistic explanation for the increased risk of AD in individuals with type 2 diabetes mellitus, as well as for the observation that tau seeds extracted from AD brains exhibit stronger prion-like activity than those generated *in vitro* from purified tau protein.

### Co-pathologies

Beyond heterologous protein cross-seeding, the pathogenesis of tauopathies is further complicated by the frequent presence of co-pathologies. In AD, Aβ deposition, as well as aggregates of TDP-43 and α-Syn, often coexist with tau pathology (Basha et al., 2026). These co-pathologies have been shown to significantly influence tau seeding activity and are associated with more rapid cognitive decline. Therefore, a comprehensive understanding of tauopathies must account for the impact of these concurrent pathological processes.

#### Aβ

Aβ pathology plays a pivotal role in accelerating tau propagation. Accumulating evidence indicates that Aβ deposition creates a permissive environment that facilitates the seeding, spread, and maturation of tau pathology. Computational modeling has demonstrated that Aβ and neuronal activity synergistically promote tau seeding in the entorhinal cortex (Alexandersen et al., 2026). In non-human primates, intracerebral injection of tau seeds derived from AD brains triggered tau propagation; however, the development of NFTs, increased CSF total-tau, and synaptic degeneration occurred only when oligomeric Aβ was co-administered. This suggests that Aβ enhances the maturation and toxicity of tau pathology rather than its initial spread (Darricau et al., 2024). Mechanistically, Aβ fibrils may induce microglial upregulation of the heparan sulfate proteoglycan Glypican 4, which, in turn, promotes neuronal uptake and seeding of tau aggregates in trans (Holmes et al., 2025). Collectively, these findings establish that Aβ pathology not only co-occurs with tauopathy but also actively potentiates tau seeding and propagation, providing a strong rationale for combination therapies targeting both pathologies in AD.

#### Alpha-synuclein

Misfolded α-syn aggregates (α-syn seeds) have been shown to co-occur with tau pathology in AD (Manca et al., 2023; Jonaitis et al., 2025). Moreover, CSF α-syn seed activity is associated with AD progression and cognitive decline (Tosun et al., 2024), underscoring the clinical significance of α-syn as a co-pathology in AD. Beyond epidemiological and biomarker correlations, mechanistic studies have revealed direct molecular interactions between these two pathogenic proteins. Notably, α-syn and tau can form hybrid fibrils with enhanced seeding potency and can potentiate each other’s phosphorylation in cellular models (Zhan et al., 2025). Their bidirectional crosstalk is further supported by *in vivo* evidence: tau accelerates α-syn aggregation and spreading in PD (Pan et al., 2022), while co-injection of α-syn seeds and tau seeds in non-human primates results in an additive effect on nigral degeneration, suggesting that α-syn pathology may exacerbate tau-mediated neurotoxicity (Darricau et al., 2025). Collectively, these findings position α-syn and tau co-pathology as a critical determinant of AD progression and clinical heterogeneity, highlighting the need for integrated therapeutic strategies that target both proteinopathies simultaneously.

#### TDP-43

Patients with AD and concurrent TDP-43 pathology experience more rapid disease progression, suggesting that the coexistence of multiple misfolded protein seeds may synergistically exacerbate neuronal vulnerability (Rahman et al., 2025). However, two lines of evidence have produced seemingly divergent findings regarding the impact of TDP-43 on tau seeding. In a clinicopathological study, AD cases with comorbid limbic-predominant age-related TDP-43 encephalopathy neuropathological changes (LATE-NC) showed greater burdens of pretangles and NFTs, elevated brain tau phosphorylation, and a positive correlation between TDP-43 burden and Braak stages (Tomé et al., 2023). Functionally, extracts from AD (LATE-NC^+^) brains enhanced tau seeding in a tau biosensor cell line and induced more severe hippocampal tau seeding in TDP-43^*A*315*T*^ transgenic mice, although no *de novo* TDP-43 aggregation was observed (Tomé et al., 2023). By contrast, using purified proteins and cell-based assays, Simonetti et al. found that direct interaction between TDP-43 and tau promoted their co-condensation, but TDP-43 suppressed tau fibril formation and hindered tau seeding in biosensor cells (Simonetti et al., 2025). Furthermore, insoluble fractions from AD brains containing tau/TDP-43 co-aggregates displayed reduced tau seeding, yet potently seeded TDP-43 aggregation in reporter cells (Simonetti et al., 2025). Thus, the relationship between TDP-43 and tau seeding appears to be context-dependent, with outcomes varying between complex pathological tissues and simplified molecular systems. The precise mechanisms underlying these contrasting observations and their relevance to human disease remain to be elucidated.

As highlighted in a comprehensive review by Basha et al., age-related neurodegenerative diseases–exemplified by AD–are increasingly recognized not as linear “one protein–one disease” entities, but as multifactorial proteinopathies involving co-aggregation and intricate interaction networks among multiple misfolded proteins, including tau, α-synuclein, Aβ, and TDP-43 (Basha et al., 2026).

## Challenges and caveats in tau seeding research

Overall, existing *in vitro*, cellular, and animal models have elucidated the core sequence of tau seed conformation, uncovered conformational and seeding activity differences among distinct tau strains, delineated the aggregation properties conferred by various *MAPT* mutations and tau isoforms, and identified multiple PTMs, LLPS, and extrinsic factors that modulate tau aggregation. Nevertheless, it must be acknowledged that these models are highly simplified and differ substantially from the complex pathological environment of the human brain.

First, in *in vitro* aggregation assays, although they allow direct assessment of the aggregation propensity of tau isoforms, the concentrations of tau or exogenous regulatory factors–such as cofactors and molecular chaperones–are often far above physiological levels, and their physiological relevance therefore requires careful validation.

Second, seeding experiments in cellular models are predominantly performed in tumor-derived cell lines (e.g., HEK293 biosensors) that overexpress mutant tau or truncated fragments. These systems are valued for their high sensitivity, reproducibility, and amenability to high-throughput screening. Nevertheless, these cell lines lack the structural and metabolic machinery of neurons, and the measured “seeding activity” reflects a simplified ability to template aggregation rather than the authentic propagation of tau seeds in the brain, which involves neurons and a range of glial cell types.

Third, limitations of current animal models remain a significant concern. The most widely used models–tau transgenic mice–often fail to accurately recapitulate the region-to-region propagation of tau seeding observed in the human brain. Tau seeding models, involving the injection of recombinant tau filaments or AD brain–derived pathological tau into recipient mice (such as htau, tau transgenic, or Aβ model mice) (Li et al., 2023a; Chen et al., 2024; Andreo-Lopez et al., 2025; Kosa et al., 2025), offer certain advantages: the former can rapidly induce pathology that mimics human disease processes, while the latter facilitate investigation of the interplay between Aβ and tau pathologies. However, intrinsic differences between murine and human physiological environments–including species-specific protein expression, PMT machinery, and cellular milieu–may confound data interpretation and limit translational relevance. Notably, recent studies have shown that intracerebral injection of AD-derived tau seeds into non-human primates (NHPs) induces tau pathology that closely mirrors Braak-staged progression, benefiting from greater neuroanatomical and physiological homology to humans (Darricau et al., 2023; Darricau et al., 2024; Datta and Arnsten, 2025). Still, the use of NHP models is constrained by practical limitations, such as extended experimental timelines and high costs, restricting their applicability for large-scale or high-throughput studies. Consequently, the development of models that more faithfully emulate human disease–coupled with rigorous cross-validation against clinical data and the selection of the most translationally relevant systems for each specific question–will be essential for advancing the field and consolidating current conceptual frameworks.

In addition to the aforementioned experimental system differences, the reproducibility of tau seeding assays is further compromised by multiple factors. (1) Seed source variability: tau seeds derived from patient brains differ in disease stage, regional distribution of pathology, and the degree of comorbid pathologies, which may confound direct activity comparisons. (2) Extraction protocol heterogeneity: inter-laboratory variations in centrifugation speeds, sonication parameters, and detergent concentrations can significantly influence the efficiency of seed extraction and apparent seeding potency. (3) Substrate selection: the use of different tau substrates–including full-length protein, isoform-specific tau, truncated fragments, site-directed mutants, or tau with distinct PTM profiles–can yield divergent seeding results. These variables collectively undermine the comparability of findings across studies and represent major contributors to the reproducibility crisis in the field. Thus, we suggest that readers remain vigilant to methodological heterogeneity across studies and interpret the results within the specific experimental framework in which they were generated.

While this review focuses on factors driving tau seed formation, it is important to note that the subsequent propagation of tau pathology is regulated by additional mechanisms. In particular, processes such as cellular uptake (Zhao et al., 2021), intracellular processing and metabolism (Renna et al., 2026), release into the extracellular space (Goedert et al., 2017), clearance (Hung, 2026), neuron-glia crosstalk (Zhou et al., 2023), and inflammatory modulation (Johnson and Lukens, 2025) all contribute to the complexity of tau pathology.

## Potential application of tau seeding activity as a biomarker for AD

The National Institute on Aging and the Alzheimer’s Association (NIA-AA) have proposed the ATN biomarker framework to systematically categorize the core pathological processes of AD into three main components: “A” for amyloid-β, “T” for pathological tau, and “N” for neurodegeneration. Among these, the “T” component specifically refers to pathological tau, which is currently evaluated primarily using cerebrospinal fluid (CSF) markers, such as pTau181 and total tau, exemplified by assays like Elecsys^®^ AD CSF. Emerging evidence suggests that measuring tau seeding activity–a functional indicator of pathogenic tau–could provide valuable complementary information for the “T” component within the ATN framework. For instance, Houzé et al. utilized biosensor cells to detect tau seeding activity in the CSF of AD patients and observed that individuals with high or intermediate seeding activity experienced significantly more rapid cognitive decline compared to those with low seeding activity. This finding highlights its potential as a predictor of disease progression rate (Houzé et al., 2025). Moreover, direct assessment of plasma pTau seeding activity may offer a more accurate reflection of early tau pathology than pTau217 concentration alone (Hanseeuw et al., 2026). Beyond CSF and plasma, tau-seeding amplification assays (tau-SAA) performed on skin biopsies have demonstrated the ability to reliably discriminate among various tauopathies–PSP, CBD, PD, and multiple system atrophy (MSA)–based on the biochemical and morphological features of the assay products (Dellarole et al., 2024; Wang et al., 2024). In summary, quantitative analysis of tau seeding activity shows great promise as a crucial tool for early AD diagnosis, therapeutic monitoring, and precise subtyping of neurodegenerative diseases.

## Current therapeutic strategies targeting tau protein

### Active immunotherapy

Immunotherapy is currently the largest and most rapidly advancing category of tau-targeting therapies (Table 1) and is divided into two types: active and passive immunization. Active immunization involves vaccination–typically using specific antigenic fragments of the tau protein–to stimulate the patient’s own immune system to generate antibodies against pathological tau.

**TABLE 1:** Tau-targeting therapeutics in clinical development.

Type	Name	Target	Clinical trial	Sponsor
Active immunization	AADvac1 (Cullen et al., 2024; Kovacech et al., 2024)	Tau 294–305	Phase 2	Axon Neuroscience
Active immunization	AV-980R/A (Hovakimyan et al., 2022)	Tau 2–18	Phase 1	Nuravax
Active immunization	JNJ-64042056 (Sol et al., 2025)	Tau 393–408 (pS396/pS404)	Phase 2	AC Immune, Janssen
Passive immunization	ABBV-227	Undisclosed (proline-rich region, phosphorylated epitope; blocks tau propagation)	Phase 1	AbbVie
Passive Immunization	ADEL-Y01	Tau 275–286 (Ac-K280)	Phase 1	ADEL, Oscotec
Passive immunization	APNmAb005	Undisclosed (oligomeric tau)	Phase 1	Aprinoia Therapeutics
Passive immunization	Bepranemab (Buchanan et al., 2026)	Tau 235–250	Phase 2	Roche, UCB
Passive immunization	BMS-986446 (Buchanan et al., 2026)	R1, R2, and R3 repeats	Phase 2	BMS, Prothena
Passive immunization	Etalanetug (Rawal et al., 2025)	Tau 299–303 and 362–366	Phase 2/3	Eisai
Passive immunization	Lu AF87908	Tau 386–408 (pS396)	Phase 1	Lundbeck
Passive immunization	MK-2214	Tau pS413	Phase 2	Merck
Passive immunization	VY7523	Undisclosed (C-terminus of tau; inhibited the spread of pathological tau)	Phase 1	Voyager
RNA-based therapy	ALN-5288	Undisclosed	Phase 1	Alnylam
RNA-based therapy	Diranersen	Undisclosed	Phase 2	Biogen, IONIS
RNA-based therapy	LY3954068	Undisclosed	Phase 1	Lilly
RNA-based therapy	NIO752	Undisclosed	Phase 3	Novartis
Aggregation inhibitor	AS-S603	dissociate amyloid and tau aggregates	Phase 1	Amyloid Solution
Aggregation inhibitor	HMTM	Inhibit tau aggregation	Phase 3	TauRx
Aggregation inhibitor	OLX-07010 (Davidowitz et al., 2025)	inhibit tau self-association	Phase 1	Oligomerix
AUTOTAC	ATB2005A (Lee et al., 2024)	AUTOTAC that degrades tau aggregates	Phase 1	Autotac Bio
Kinase inhibitor	Lithium (Gildengers et al., 2026)	GSK3 inhibitor, inhibit tau phosphorylation	Phase 4	University of Pittsburgh

In patients with mild AD and elevated baseline plasma p-tau217 levels, treatment with the active immunotherapy AADvac1 was safe and significantly reduced the accumulation rate of plasma neurofilament light chain (NfL) and glial fibrillary acidic protein (GFAP). Although the Clinical Dementia Rating – Sum of Boxes (CDR-SB) and the Alzheimer’s Disease Cooperative Study – Activities of Daily Living scale for Mild Cognitive Impairment, 18-item version (ADCS-ADL-MCI-18) favored the treatment group, the differences did not reach statistical significance. Notably, in the subgroup identified as both amyloid- and tau-positive, AADvac1 demonstrated an antibody-dependent slowing of AD-related clinical decline, supporting further evaluation in larger trials (Cullen et al., 2024; Kovacech et al., 2024).

Additionally, JNJ-64042056 (ACI-35.030), which targets a phospho-tau peptide from the C-terminal region of the tau protein (Tau393-408 [pS396/pS404]), demonstrated in a Phase 2 trial that it induces a rapid, durable, and selective antibody response against pTau in humans (Sol et al., 2025).

### Passive immunotherapy

Passive immunotherapy involves the direct administration of exogenous, pre-manufactured monoclonal antibodies to rapidly neutralize or clear pathological tau (Li et al., 2022). A Phase 1 trial of Etalanetug (E2814) demonstrated that it was safe and well-tolerated following both single and multiple infusions (Rawal et al., 2025). CSF concentrations of Etalanetug between 100 and 200 ng/mL achieved 82.1% saturation binding of tau299 and 64.9% saturation binding of tau354, indicating an adequate safety and immunogenicity profile in healthy adults. Meanwhile, Bepranemab (UCB0107), which targets the mid-region of the tau protein (Buchanan et al., 2026), showed an acceptable safety profile in Phase 2 clinical trials.

### RNA-based therapy

RNA-based therapies utilize antisense oligonucleotides (ASOs) or RNA interference technology to directly inhibit MAPT gene expression, thereby reducing tau protein production and subsequently slowing tau aggregation and disease progression. Diranersen is the first tau-targeting ASO to enter clinical trials for mild AD (Lozupone et al., 2023), demonstrating favorable brain distribution (Cook et al., 2025). In the high-dose group, treatment with Diranersen led to a greater than 50% reduction from baseline in CSF total tau and p-tau181 (Edwards et al., 2023), and showed a trend toward sustained slowing of decline in cognitive function and global assessments (Shulman et al., 2026), supporting further development.

### Aggregation inhibitors

These small-molecule compounds directly inhibit tau protein aggregation, fibril formation, and seeding activity. Their potential to inhibit the pathological progression of AD is also being explored clinically. The most representative drug in this class is hydromethylthionine mesylate (HMTM), a methylene blue derivative and the most advanced small-molecule tau aggregation inhibitor currently in Phase 3 clinical trials. Previous large clinical trials of HMTM did not meet their primary endpoints. However, a recent analysis using an “external control group” dataset found that the treatment group showed significant improvements in ADAS-Cog13, whole brain volume, and CDR-SB, as well as reductions in serum NfL and p-tau217, and a slowing of cerebral gray matter atrophy (Schelter et al., 2026; Wischik et al., 2026).

### Other therapies

Lithium, conventionally used to treat mood disorders, has been investigated in a Phase 4 clinical study for AD due to its ability to reduce tau phosphorylation by inhibiting GSK3 activity. However, a clinical trial conducted at the University of Pittsburgh School of Medicine from February 2018 to August 2024, with a 2-year follow-up, found that primary endpoints–including cognitive performance, hippocampal volume, cortical gray matter volume, and brain-derived neurotrophic factor (BDNF)–did not achieve statistical significance (Gildengers et al., 2026).

Additionally, tau-targeted degradation drugs such as ATB2005A (Lee et al., 2024)–an autophagy-targeting chimera (AUTOTAC) that recognizes and degrades tau aggregates via a modified emrusolmin moiety–are currently undergoing active clinical investigation.

Beyond the strategies previously discussed, additional tau-targeted modalities–such as Proteolysis-Targeting Chimeras (PROTACs) (Wang et al., 2021; Zhang J. et al., 2024) and inhibitors of tau uptake/propagation (e.g., heparinoids) (Wang et al., 2021)–have also been reported. However, these approaches remain at the preclinical stage, with none having advanced to clinical trials thus far.

### Combination therapies

Combination strategies that target tau pathology alongside other therapeutic modalities–particularly anti-Aβ antibodies–are currently under evaluation in ongoing clinical trials. For example, the active tau immunotherapy AADvac1 is being tested in combination with the anti-Aβ antibody Donanemab (ClinicalTrials.gov ID: NCT07167966). Likewise, the anti-tau antibody Etalanetug (E2814) is being investigated in conjunction with Lecanemab, both in dominantly inherited AD (NCT05269394) and in a separate study of early sporadic AD (NCT06602258). Although clinical outcomes from these trials are not yet available, these combination regimens are anticipated to yield valuable insights into the advantages and challenges of dual-target approaches, ultimately informing the development of more effective, multi-modal therapeutic strategies for AD.

### Challenges in tau-targeting therapies for AD

Despite substantial efforts to develop tau-targeting therapies for AD, the field has faced considerable setbacks and obstacles. The leading tau aggregation inhibitor to reach Phase 3 clinical trials, HMTM, failed to demonstrate a difference in clinical endpoints. This shortcoming was largely attributed to a flawed placebo design: the control agent, methylthioninium chloride, also delivers the active HMT moiety (van Maanen et al., 2026). Additionally, HMTM’s efficacy appears to be diminished–or even negated–when administered alongside commonly used cholinesterase inhibitors (Santos et al., 2022).

Multiple anti-tau antibodies, such as Tilavonemab and Gosuranemab, have also proven ineffective in slowing cognitive decline. A major challenge is their inadequate access to the pathological site of action. While these antibodies demonstrate high target engagement in the CSF–up to 92% and 99%, respectively–this engagement plummets to only 1–3% within the synaptic cleft, resulting in a reduction of oligomeric tau neuronal uptake to less than 0.1% (Geerts et al., 2023). Furthermore, postmortem analysis of Gosuranemab-treated patients revealed an associated glial response in which tau was sequestered within astrocytic lysosomes rather than being effectively cleared (Kim et al., 2021). Moreover, the focus on N-terminal tau epitopes may further compromise efficacy, as truncated tau species are prevalent in diseased brains.

Other investigational agents have failed due to mechanism-based toxicity. For example, Ceperognastat–a potent, orally bioavailable, and brain-penetrant O-GlcNAcase inhibitor designed to reduce tau phosphorylation–proved not only clinically ineffective but potentially harmful, due to intrinsic synaptic toxicity (Meade et al., 2026).

Thus, the successful translation of tau-targeting therapies is impeded by a complex array of translational barriers–including control arm design, drug delivery limitations, epitope selection, off-target effects and associated toxicities, and concerns regarding long-term safety. This reality underscores the need for a more holistic and integrated approach to these challenges during the drug design phase, with the goal of improving the prospects for clinical success.

In summary, while numerous therapeutic strategies targeting tau protein have been developed for AD, none has yet achieved its primary endpoint in Phase III clinical trials, making direct efficacy comparisons currently unfeasible. Of these approaches, passive immunotherapy and RNA-based therapies have shown the most promising biomarker responses; however, their clinical benefits remain to be validated in subsequent studies. The efficacy of aggregation inhibitors is still inconclusive, and clinical data supporting tau degradation–promoting strategies or anti-seeding approaches in human populations are presently lacking. Consequently, there is an urgent need for larger, more comprehensive clinical trials featuring standardized endpoints and direct head-to-head comparisons with existing AD therapies to thoroughly evaluate both safety and efficacy, thereby guiding clinical decision-making.

## Perspectives

Despite remarkable progress, several challenges and unanswered questions remain, shaping the future agenda for tau seeding research and the broader understanding of AD. A central unresolved question concerns the mechanisms underlying selective neuronal vulnerability to tau pathology–why specific populations such as entorhinal cortex layer II neurons (Alexandersen et al., 2026), basal forebrain cholinergic neurons (Berry and Harrison, 2023), and certain cortical subtypes (Broekaart et al., 2025) are disproportionately affected while others remain resilient. Elucidating this differential susceptibility will require integrating multi-omics analyses of both vulnerable and resistant neurons, allowing for a detailed dissection of the interplay between cell-intrinsic factors (e.g., ion channel composition, metabolic profiles, proteostatic capacity) and cell-extrinsic influences (e.g., glial interactions, synaptic connectivity, local inflammation).

In parallel with these mechanistic investigations, a key priority is the development of animal models that more accurately recapitulate the propagation of tau pathology seen in human sporadic AD. This entails incorporating authentic human tau isoforms, relevant seed conformations, and age-associated comorbidities to better mirror disease progression. The properties of tau seeds themselves also demand deeper exploration–particularly how their structural conformations and PTM profiles confer tropism for specific neuronal subtypes. Insights here could guide the creation of targeted diagnostics and therapies capable of selectively neutralizing pathogenic seeds while preserving physiological tau functions.

Furthermore, the phenomenon of trans-seeding–whereby distinct pathological proteins such as tau, Aβ, and α-synuclein cross-seed and mutually promote aggregation–merits investigation for its broader impact on AD pathology. Understanding these interactions will be crucial in determining the feasibility and optimal timing for combination therapies. Ultimately, addressing these knowledge gaps holds the promise not only of advancing fundamental insights into tau pathobiology, but also of laying the groundwork for precision medicine approaches in AD and related tauopathies.

## Conclusion

The past 5 years have transformed our understanding of tau seeding from a conceptual hypothesis to a mechanistically defined process with multiple druggable targets, and continued integration of structural biology, cellular models, animal studies, and clinical trials will be essential for advancing effective tau-targeted therapies for AD and related tauopathies.

## Footnotes

1
www.alzforum.org
